# Automatic facial coding predicts self-report of emotion, advertisement and brand effects elicited by video commercials

**DOI:** 10.3389/fnins.2023.1125983

**Published:** 2023-05-02

**Authors:** T. Tim A. Höfling, Georg W. Alpers

**Affiliations:** Department of Psychology, School of Social Sciences, University of Mannheim, Mannheim, Germany

**Keywords:** automatic facial coding, action units (AU), FACS, facial expression, emotion, advertisement, brand, semiparametric additive mixed models

## Abstract

**Introduction:**

Consumers’ emotional responses are the prime target for marketing commercials. Facial expressions provide information about a person’s emotional state and technological advances have enabled machines to automatically decode them.

**Method:**

With automatic facial coding we investigated the relationships between facial movements (i.e., action unit activity) and self-report of commercials advertisement emotion, advertisement and brand effects. Therefore, we recorded and analyzed the facial responses of 219 participants while they watched a broad array of video commercials.

**Results:**

Facial expressions significantly predicted self-report of emotion as well as advertisement and brand effects. Interestingly, facial expressions had incremental value beyond self-report of emotion in the prediction of advertisement and brand effects. Hence, automatic facial coding appears to be useful as a non-verbal quantification of advertisement effects beyond self-report.

**Discussion:**

This is the first study to measure a broad spectrum of automatically scored facial responses to video commercials. Automatic facial coding is a promising non-invasive and non-verbal method to measure emotional responses in marketing.

## Introduction

Consumer neuroscience promises a better understanding of consumers’ emotions and attitudes with objective measures. Emotions play a central role in attitude formation (Ito and Cacioppo, 2001), information processing (Lemerise and Arsenio, 2000; Fraser et al., 2012), and decision-making in general (Slovic et al., 2007; Pittig et al., 2014). Hence, a direct measurement of emotional responses with advanced technologies might be key to understanding consumers’ behavior and decisions (Ariely and Berns, 2010; Solnais et al., 2013). In particular, emotions play a central role in marketing communications, such as video commercials to elicit desired advertisement and brand effects (Achar et al., 2016). Correspondingly, advertisements influence customers’ perception of brands which potentially moderates their purchase decisions and behaviors (Plassmann et al., 2012). Furthermore, consumers’ expectancies about a particular brand have a strong psychological impact because they can modulate consumption experience (McClure et al., 2004; Shiv et al., 2005). Neural activation patterns elicited by video commercials were previously investigated to either predict advertisement effectiveness beyond self-report with fMRI (Berns and Moore, 2012; Falk et al., 2012) and electroencephalogram (EEG) (Dmochowski et al., 2014; Boksem and Smidts, 2015) or to display latent emotional processes on a continues basis (Ohme et al., 2010; Vecchiato et al., 2011). However, such measures either require obtrusive research settings (fMRI tubes) or sensors attached to the scalp (EEG) and are, thus, not entirely non-invasive.

### Facial expression and emotion

Besides brain activity, emotional experiences also induce affective expressions (Sander et al., 2018; Scherer and Moors, 2019). Facial expression is the most investigated and predictive aspect of emotional expressions (Scherer and Ellgring, 2007; Plusquellec and Denault, 2018). In comparison to measures of brain activity, facial expressions responses are typically video-based, which requires no measurement preparation or application, can be obtained in ecologically valid environments, and is even applicable to online research. In order to capture emotionally relevant information from the whole face, researchers have heavily relied on observation techniques such as the Facial Action Coding System (FACS) to score intensity estimates of single facial movements called Action Units (AU) (Ekman et al., 2002; Mauss and Robinson, 2009). FACS is an extensive coding manual which allows for a very detailed description of facial responses through the combination of AU and shows good to excellent inter-rater reliabilities (Sayette et al., 2001). However, human FACS coding results in low scaling resolution of AU intensities and it is very time consuming (Schulte-Mecklenbeck et al., 2017).

Although there are several important theories that explain specific aspects of emotional facial expressions [for an overview, see Barrett et al. (2019)], there are currently only two relevant empirical approaches that map combinations of specific AUs to specific emotional states. On the one side, basic emotion theory predicts robust AU patterns that cohere with a limited amount of distinct emotion categories (i.e., joy, anger, disgust, sadness, fear, and surprise; e.g., Ekman et al., 1987). However, there is evidence for universal facial expressions beyond six basic emotions (Cordaro et al., 2018, 2020; Keltner et al., 2019; Cowen et al., 2021). Moreover, there are spontaneous emotional responses that are be much more variable and less universal for a distinct AU pattern (Durán and Fernández-Dols, 2021; Le Mau et al., 2021; Tcherkassof and Dupré, 2021). On the other side, componential process theory predicts several appraisal dimensions that elicit specific AU combinations like valence, novelty, and control (Sander et al., 2018; Scherer et al., 2018, 2021).

Although electromyography (EMG) research extensively measured corrugator and zygomaticus activity to approximate a valence dimension (e.g., Höfling et al., 2020), the investigation of other components in this theory is still preliminary. Hence, there is currently no consensus about the link between meaningful AU combinations regardless of the assumption of dimensional or categorial underlying emotional processes.

### Validity of AFC

Recent advances in technology have enabled the measurement of facial expressions to obtain emotion-associated parameters through automatic facial coding based on machine-learning (AFC; Pantic and Rothkrantz, 2003; Cohn and Sayette, 2010). AFC parameters include separate facial movements, such as Action Units derived from the Facial Action Coding System on the one side and integrated “emotion-scores” such as joy or anger estimations, on the other side. There is evidence that AU parameters estimated by AFC correspond with estimates of human FACS raters between 71 and 93%, depending on the specific measurement system (Bartlett et al., 1999; Tian et al., 2001; Skiendziel et al., 2019; Seuss et al., 2021). Moreover, AFC classifies basic emotional facial expressions with impressive accuracy in prototypical facial expressions in pictures (Lewinski et al., 2014a; Lewinski, 2015; Beringer et al., 2019; Küntzler et al., 2021) as well as videos (Mavadati et al., 2013; Yitzhak et al., 2017; Calvo et al., 2018; Krumhuber et al., 2021).

While attempts to validate this innovative technology mainly focused on highly standardized and prototypical emotional facial expressions in the past, the number of validation studies that approximate more naturalistic or spontaneous facial expressions is still preliminary. AFC is less accurate in the detection of less intense and more naturalistic expressions (Büdenbender et al., 2023), which is also a commonly observed pattern in human emotion recognition (Krumhuber et al., 2021). Some studies find evidence that AFC can be transferred to the facial expressions of naïve participants that mimic emotional facial expressions in a typical laboratory setting (Stöckli et al., 2018; Sato et al., 2019; Höfling et al., 2022). To a limited extent, AFC detects highly unstandardized emotional facial expressions of professional actors depicted in movies (Küntzler et al., 2021). Furthermore, AFC can also track spontaneous emotional responses toward pleasant scenes, where AFC parameters correlate with emotional self-report and direct measures of muscle activity with EMG (Höfling et al., 2020). However, AFC is not sensitive to very subtle emotional responses, particularly if participants are motivated to suppress or control their facial responses (Höfling et al., 2021). Taken together, there is evidence that AFC validly captures spontaneous emotional states in a typical laboratory setting, especially for pleasant emotional responses.

### AFC and advertisement

According to the affect-transfer hypothesis (Brown and Stayman, 1992), there is evidence for a relationship between consumers’ emotional responses to advertisement stimuli and the subsequently elicited advertisement and brand effects. In this model, an advertisement elicits emotional responses which influence the attitude toward the advertisement (i.e., advertisement likeability). In the following step, a favorable ad likeability leads to changes in the attitude toward the brand (brand likeability), which increases the purchase intention of products and services of a particular brand in the final step of this process model.

AFC has been used successfully to predict the effects of video commercials’ on the subsequent processes of this advertisement and brand effect framework, such as the self-reported emotional response, advertisement likeability, as well as brand likeability and purchase intention. In the domain of political influencing, AFC measures have been shown to correspond with emotional self-report of pleasant (Mahieu et al., 2019) as well as unpleasant advertisements (Fridkin et al., 2021). They are also predictive to measure intended emotional responses in an *a priori* defined target audience (Otamendi and Sutil Martín, 2020). Accordingly, AFC of smiling intensity correlates with advertisement likeability (Lewinski et al., 2014b; McDuff et al., 2014, 2015), brand likeability (Lewinski et al., 2014b), and the purchase intentions of advertised brands (Teixeira et al., 2014; McDuff et al., 2015). Furthermore, increased smiling was also found to reduce zapping behavior (Yang et al., 2014; Chen et al., 2016), decrease attention dispersion (Teixeira et al., 2012), and predict long-term attitude changes (Hamelin et al., 2017). Taken together, there is evidence that AFC of smiling predicts several steps of the advertisement and brand effects proposed by the affect-transfer hypothesis. In contrast to AFC, approaches to measure advertisement effects with human FACS were less successful (e.g., Derbaix, 1995).

### Research gaps and overview

The scientific knowledge regarding a direct link between emotional facial expression on the one side and advertisement or brand effects on the other side is very limited due to the following reasons: First, it is unclear whether facial expressions significantly predict advertisement and brand effects beyond relevant precursor self-report dimensions. Such quantification is only possible if statistical models of facial expression parameters are compared with models that control for relevant self-report, which has not been investigated in previous research. Second, relevant research mainly used integrated parameters for joy or entertainment, relying almost exclusively on measurements of smiling (AU12). Reporting such integrated scores serves a lower level of scientific transparency in comparison to a description with AU terminology because it is largely unknown how AFC classifiers are trained and, correspondingly, how integrated parameters are estimated. As an additional consequence, it is unclear how different AUs that are relevant for emotional facial expressions ensembles to predict advertisement and brand effects of video commercials’ effectiveness beyond smiling. Third, there is no consensus on whether the degree of amusement or entertainment in video commercials shows a linear or non-linear relationship on branding effects: While Lewinski et al. (2014b) report a weak linear relationship between AFC of smiling and brand effects, Teixeira et al. (2014) report an inverted u-shaped relationship between AFC of smiling and brand effects. Finally, there is no study available that investigates the relationship between emotional facial expressions and all relevant steps of advertisement and brand effects according to the affect-transfer hypothesis in a within-subject study design.

**FIGURE 1:**
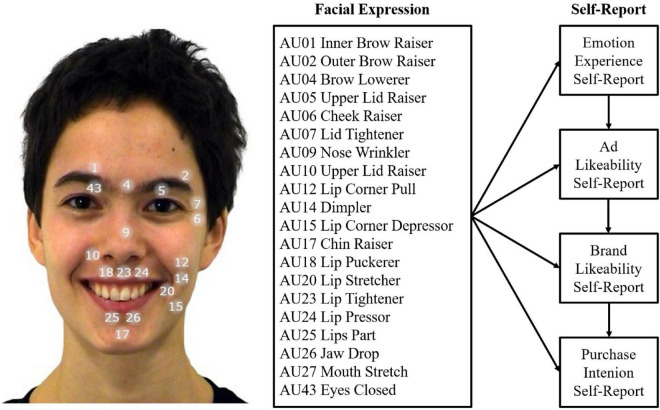
Overview of the measured Action Units and hypothesized relations to self-report ratings. Depicted is a happy facial expression from the ADFES inventory (model F04; van der Schalk et al., 2011).

In order to close these existing research gaps, we investigated the predictive value of facial expressions while viewing video commercials to forecast all subsequent components of the affect-transfer model of advertisement and brand effects: emotion ratings, ad likeability, changes between pre- and post-measurements of brand likeability, and purchase intention. To this end, we broaden the spectrum of analyzed movements in the face to 20 AU that can be measured with a state-of-the-art AFC algorithm to predict relevant outcome criteria (see Figure 1 for an overview of measured AUs and self-report ratings). In addition, we aim to determine non-linearities between facial expressions and self-report with semi-parametric additive mixed models that can account for non-linear effects (Wood, 2006; Wood et al., 2013; Wood and Scheipl, 2020). We expect strong relationships between self-reported emotion ratings and advertisement likeability and changes in brand likeability as well as purchase intention. In order to collect data from a variety of industries and emotional content, we established different stimulus groups which only differed in terms of advertisement videos and corresponding brand stimuli. This is the first study that investigates the relationship between emotional facial expressions measured by artificial intelligence and all relevant components of advertisement and brand effectiveness proposed by the affect-transfer framework.

## Materials and methods

### Participants

A total of 257 volunteers were randomly assigned to one of eight stimulus groups. General exclusion criteria were age under 18, acute psychoactive medication use, acute mental disorder episode, severe somatic disease, and wearing glasses. Participants with corrected-to-normal vision were asked to wear contact lenses during the experiment. After visual inspection of the analyzed videos, data from 38 participants were excluded because of face detection problems of the AFC software (e.g., partially covered face with the hand, scarfs, and other accessories or large body movements that lead to false face detection). Hence, we only used data from participants with good recording and analysis quality, resulting in an overall sample of 219 (115 females) participants of mainly Caucasian descent. Age ranged from 19 to 58 years (*M* = 23.79, *SD* = 5.22). All participants received compensation of either 8€ or student course credit, and they signed informed consent before the data collection. University Research Ethics Committee approved the experiment.

### Questionnaires

Participants filled in various questionnaires to compare relevant states and traits related to emotional responsiveness and expressivity to ensure comparability between experimental groups. After a socio-demographic questionnaire (e.g., gender, age, and educational level), the Social Interaction Anxiety Scale (SIAS; Stangier et al., 1999), the State-Trait Anxiety Inventory (STAI State and STAI Trait; Laux et al., 1981), the Positive-and-Negative-Affect-Schedule (PANAS PA and PANAS NA; Krohne et al., 1996), the Self-Rating Depression Scale (SDS; Zung, 1965), the Berkley Expressivity Questionnaire (BEQ; Mohiyeddini et al., 2008), and the Behavioral Inhibition and Activation Scale (BIS and BAS; Strobel et al., 2001) were administered before starting the main experiment.

### Study design and procedure

Following informed consent and completion of the questionnaires, participants were seated in front of a computer screen. In order to collect data from a variety of industries and emotional content, we established eight different stimulus groups to which participants were randomly assigned. These groups only differed in terms of advertisement videos and corresponding brand stimuli; all other aspects remained constant between groups. Before the main experiment started, participants were instructed to maintain a neutral facial expression for 10 s, which was later utilized to calibrate the AFC analysis individually. The main experiment comprised three experimental blocks: pre-evaluation of brands, viewing video advertisements and evaluation, and post-evaluation of brands.

The pre- and post-evaluations of brands were set up identically, and hence, effects on brands elicited by the video advertisement can be traced by pre-post rating changes. The logo of the eight advertised brands was also presented in both brand evaluation blocks, each started with a 1 s fixation cross. The presentation duration of the brand logos lasted until participants decided when to proceed with several ratings of a specific brand by pressing the space bar. All scales were presented as a nine-point semantic differential. Participants rated their familiarity with the brand (1 = familiar and 9 = unfamiliar), brand likeability (1 = like and 9 = dislike; 1 = good and 9 = bad), and brand purchase intention (1 = probable purchase and 9 = unprobeable purchase; 1 = purchasing definitely and 9 = purchasing definitely not) after the brand presentation. All advertisements were indicated by a 1 s fixation cross in the advertisement block. After watching a particular video, each video was rated on nine-point semantic differentials containing advertisement familiarity (1 = familiar and 9 = unfamiliar) and advertisement likeability (1 = like and 9 = dislike; 1 = good and 9 = bad). In addition, participants rated how they felt during each video presentation on several one-item emotion scales (i.e., joy, sadness, anger, disgust, fear, surprise; 1 = strong emotion and 9 = no emotion) immediately after the video presentation. All scales were inverted to improve the readability of the results. If two items were used to measure a construct (i.e., advertisement likeability, brand likeability, brand purchase intention), the average of both items would have been calculated. Internal consistencies were excellent for these three scales (Cronbach’s α > 0.90).

### Stimulus material

Each group watched eight different commercials and corresponding brand logos in randomized order. The video material was selected from the list of award-winning commercials at the Cannes Lions International Festival of Creativity between 2016 and 2017. The videos were counterbalanced between groups in terms of video duration, familiarity with the brand, familiarity with the video, and emotional content (see also Section “Results”). Appendix 1 contains all commercial video advertisements sorted by groups. Two other non-commercial video advertisements per group were presented but not included in the analysis because the brand ratings did not apply to the non-commercial section.

### Apparatus and measurements

High-precision software (E-Prime; Version 2.0.10; Psychology Software Tools, Pittsburgh, PA, USA) was used to run the experiment. Stimuli were shown centrally on a 19-inch monitor with a resolution of 1,024 × 768, approximately 70 cm away from the participant. Optimal illumination with diffused frontal light was maintained throughout. Videos of participants’ faces were recorded with a Logitech HD C615 web camera placed above the computer screen. Videos were processed off-line with FaceReader Software (FR; Version 7.0, Noldus Information Technology) and further analyzed with Observer XT (Version 12.5, Noldus Information Technology). Furthermore, Observer XT synchronized stimulus onset trigger from E-Prime and the video recordings. FR is a visual pattern classifier based on deep learning approaches and extracts visual features from videos frame by frame. In accordance with neuro-computational models of human face processing (Dailey et al., 2002; Calder and Young, 2005), FR detects facial configurations in the following steps (Van Kuilenburg et al., 2005, 2008): (1) The *Cascade classifier algorithm* finds the position of the face (Viola and Jones, 2004). (2) Face textures are normalized and the *active appearance model* synthesizes a digital face model representing facial structure with over 500 location points. (3) Compressed distance information is then transmitted to an artificial neural network. (4) Finally, the artificial neural network connects these scores with relevant emotional labels through supervised training with over 10,000 samples (pictures) of emotional faces to classify the relative intensity of a given facial configuration. As a result, FR estimates activity of 20 AU, which includes AU01 Inner Brow Raiser, AU02 Outer Brow Raiser, AU04 Brow Lowerer, AU05 Upper Lid Raiser, AU06 Cheek Raiser, AU07 Lid Tightener, AU09 Nose Wrinkler, AU10 Upper Lid Raiser, AU12 Lip Corner Pull, AU14 Dimpler, AU15 Lip Corner Depressor, AU17 Chin Raiser, AU18 Lip Puckerer, AU20 Lip Stretcher, AU23 Lip Tightener, AU24 Lip Pressor, AU25 Lips Part, AU26 Jaw Drop, AU27 Mouth Stretch, and AU43 Eyes Closed. The estimated parameter of each AU ranges from 0 to 1. FR measures were calibrated per participant based on the baseline measurement at the beginning of the experiment. “East-Asian” or “elderly” face models were presented instead of the general face model as recommended by the user manual. For the duration of each video, the mean and peak activity for all AUs were exported and analyzed.

### Data reduction and analysis

Across all participants (*N* = 219) and commercial video stimuli (*n* = 64; eight per participant), 1,752 data points were collected on advertisements and corresponding brand ratings. Our data analysis required several steps: analysis of participant characteristics, aggregation of video- and brand-wise means for analysis of stimulus characteristics, and also to report correlations between facial expression parameters and relevant self-report ratings, as well as semi-parametric mixed additive regression models to predict emotion, advertisement and brand effects based on facial expression parameters.

First, we calculated ANOVA with the factor stimulus group (eight groups) to determine differences in participant characteristics separately for STAI State, STAI Trait, SIAS, BIS, BAS, PANAS PA, PANAS NA, BEQ, and SDS. In addition, we report differences in gender distribution in stimulus groups with the Chi-Squared test.

Second, we calculated pre-post difference scores for brand likeability and brand purchase intention and averaged relevant self-report ratings and AU parameters separately for each video and the corresponding brand. On the one side, we determined differences in stimulus characteristics based on stimulus-wise averages for the stimulus groups with ANOVA for video duration, brand familiarity (pre-rating), video familiarity, and emotion ratings (i.e., joy, sadness, anger, fear, disgust, and surprise). We applied a Greenhouse–Geisser correction for ANOVA when appropriate. Eta-squared (η^2^) is reported as an effect size for F-tests (η^2^ ≥ 0.01 small; η^2^ ≥ 0.06 medium; η^2^ ≥ 0.14 large; Pierce et al., 2004).

Third, we identified AU that showed zero or near-zero variance to exclude AU that will not contribute to the prediction of relevant outcomes. This exclusion criterion applied for eight AU, in particular, AU01 Inner Brow Raiser, AU02 Outer Brow Raiser, AU09 Nose Wrinkler, AU10 Upper Lid Raiser, AU18 Lip Puckerer, AU20 Lip Stretcher, AU26 Jaw Drop, and AU27 Mouth Stretch, which were excluded from further analysis. Further, we report spearman’s rho correlations between relevant AU and self-report based on averages for each video and the corresponding brand. Effect sizes were interpreted following Cohen (1988): *r* ≥ 0.1 small, *d* ≥ 0.3 medium, and *d* ≥ 0.5 large.

Fourth, all self-report ratings were z-transformed on the group level for better interpretability and comparability of the effects. To resolve the limitation of the one-item scale of emotion ratings for joy, z-scores are calculated based on individual participant ratings. In contrast to self-report ratings, AU variables were not transformed in any way because of their scale properties (e.g., exact zero-point) and correspondence to the intensity measurement of the Facial Action Coding System: Values from >0.00–0.16 are classified as trace (E), 0.16–0.26 as slight (D), 0.26–0.58 as pronounced (C), 0.58–0.90 as severe (B), and 0.90–1 as max intensity (A).

Fifth, as predictive models, we carried out semi-parametric additive mixed modeling with the R-package “gamm4” (Wood and Scheipl, 2020). In the first step, we developed a basic model controlling gender and stimuli as fixed factors and the participants and stimulus group as random factors. Next, we calculated main effect models for all AU separately for peak and mean activity to predict joy ratings, advertisement likeability, brand likeability change, and brand purchase intention change. Furthermore, we estimated the combined effect of AU and relevant rating scales to determine the relative predictiveness of AFC versus self-report resulting in 17 independent models. Visualization of the most substantial effect patterns is presented as smoothed effect plots with 95% confidence intervals. We report *R^2^_*adj*_*, Akaike-Information-Criterion (*AIC*), and Bayesian-Information-Criterion (BIC) as a goodness of fit indices (Burnham and Anderson, 2004). According to Cohen (1988), we interpreted the adjusted *R*^2^ ≥ 0.01 as small, *R*^2^ ≥ 0.09 as moderate, and *R*^2^ ≥ 0.25 as a large proportion of explained variance of each predictive model. A large change in model fit was interpreted by an absolute change of 10 for AIC and BIC. The significance level was always set to α = 0.05.

## Results

### Manipulation checks

#### Questionnaire group differences

Participants were randomly assigned to one of eight groups with different stimulus materials. Appendix 2 shows descriptive statistics of the questionnaires separately for the groups. There were no significant differences between groups regarding STAI State, STAI Trait, SIAS, BIS, BAS, PANAS PA, PANAS NA, BEQ, SDS, and gender distribution. Hence, no meaningful differences in participant characteristics between groups can be reported.

#### Stimulus group differences and stimulus emotion characteristics

Appendix 1 shows descriptive statistics for all advertisement videos and averaged scores of relevant brand and advertisement evaluations. Comparison of the different stimulus groups revealed no significant differences for video duration, *F*(7, 56) = 0.32, *p* = 0.944, η^2^ = 0.04, brand familiarity (pre-rating), *F*(7, 56) = 0.20, *p* = 0.985, η^2^ = 0.02, video familiarity, *F*(7, 56) = 1.62, *p* = 0.148, η^2^ = 0.17, joy, *F*(7, 56) = 1.58, *p* = 0.162, η^2^ = 0.17, sadness, *F*(7, 56) = 0.09, *p* = 0.999, η^2^ = 0.01, anger, *F*(7, 56) = 0.93, *p* = 0.489, η^2^ = 0.10, fear, *F*(7, 56) = 0.95, *p* = 0.476, η^2^ = 0.11, disgust, *F*(7, 56) = 0.91, *p* = 0.503, η^2^ = 0.10, and surprise ratings, *F*(7, 56) = 1.22, *p* = 0.299, η^2^ = 0.13. Hence, no meaningful differences in stimulus characteristics between groups were found.

Importantly, different videos elicited different emotions, *F*(5, 315) = 166.59, *p* < 0.001, η^2^ = 0.73. While participants reported generally higher amounts of joy (*M* = 5.42, SD = 1.43) and surprise (*M* = 4.33, SD = 1.01), other emotions were reported substantially less (sadness: *M* = 2.15, SD = 1.33; anger: *M* = 1.79, SD = 0.60; fear: *M* = 1.58, SD = 0.61; disgust: *M* = 1.65, SD = 0.89) and, hence, were not included in the main analysis (i.e., semiparametric models of AU activity).

### Correlations between self-reports and facial expressions

Spearman correlations based on unstandardized average values per stimulus between emotion, advertisement, brand ratings, and AU intensity for mean and peak activity over the video duration are reported in Table 1. There were strong and positive correlations between facial expression measures of AU6, AU12, and AU25 for ratings of joy as well as surprise for both mean and peak AU activity. Although feelings of surprise can be elicited by pleasant to unpleasant emotional events, the advertisements presented in this study elicited the same correlational patterns of facial activity for higher surprise and higher joy ratings. Therefore, these two emotion ratings might be confounded in the present study design, probably because the videos mainly triggered pleasant emotions. Hence, we focused on joy ratings as a self-report measure of emotion elicited by video commercials in the main analysis (i.e., semiparametric models of AU activity).

**TABLE 1:** Spearman correlations based on unstandardized average values per stimulus between self-report ratings and Action Unit (AU) activity (mean and peak).

	Joy	Surprise	Anger	Fear	Disgust	Sadness	Ad like	Brand like	Purchase intention
Ad like	**0**.**75**	–0.06	**–0**.**42**	–0.19	**–0**.**48**	0.18			
Brand like	0.29	–0.01	–0.28	–0.19	**–0**.**32**	–0.08	**0**.**33**		
Purchase intention	0.29	–0.21	–0.22	–0.18	**–0**.**40**	–0.03	**0**.**43**	**0**.**63**	
Mean AU 04	**–0**.**51**	–0.02	0.26	**0**.**44**	0.03	0.24	**–0**.**34**	–0.10	–0.16
Mean AU 05	–0.01	–0.03	–0.02	0.12	0.06	0.08	0.04	0.00	0.10
Mean AU 06	**0**.**57**	**0**.**53**	**–0**.**21**	**–0**.**45**	0.15	**–0**.**45**	0.17	0.08	0.02
Mean AU 07	**–0**.**54**	–0.12	0.27	**0**.**38**	0.13	0.16	**–0**.**33**	0.01	–0.13
Mean AU 12	**0**.**71**	**0**.**49**	**–0**.**33**	**–0**.**53**	0.03	**–0**.**46**	0.28	0.16	0.04
Mean AU 14	–0.26	0.05	0.21	0.22	–0.10	0.12	–0.20	0.09	–0.02
Mean AU 15	–0.13	0.05	**0**.**34**	0.21	0.25	0.09	–0.11	–0.06	–0.17
Mean AU 17	–0.22	0.07	0.08	**0**.**33**	0.09	0.27	–0.02	0.07	0.02
Mean AU 23	**–0**.**44**	–0.01	0.29	**0**.**38**	–0.09	0.22	–0.29	–0.15	–0.20
Mean AU 24	–0.15	0.03	0.03	0.16	–0.17	0.16	–0.06	0.10	–0.02
Mean AU 25	**0**.**50**	**0**.**50**	–0.10	**–0**.**35**	0.17	**–0**.**43**	0.10	0.05	–0.02
Mean AU 43	0.22	–0.16	–0.24	**–0**.**33**	–0.03	–0.14	0.29	0.06	0.24
Peak AU 04	**–0**.**49**	0.07	0.21	**0**.**52**	0.14	**0**.**30**	**–0**.**39**	–0.13	–0.28
Peak AU 05	0.08	0.10	–0.06	0.19	0.01	0.17	0.06	–0.03	0.02
Peak AU 06	**0**.**61**	**0**.**55**	–0.21	**–0**.**43**	0.11	**–0**.**41**	0.24	0.13	0.05
Peak AU 07	–0.27	–0.02	0.19	**0**.**34**	0.18	0.19	–0.13	–0.01	–0.18
Peak AU 12	**0**.**72**	**0**.**52**	–0.29	**–0**.**43**	0.00	**–0**.**32**	**0**.**39**	0.16	0.05
Peak AU 14	–0.17	–0.02	0.17	0.23	–0.19	0.22	–0.04	0.12	0.00
Peak AU 15	–0.04	–0.03	0.16	0.26	0.04	**0**.**31**	0.05	0.12	0.05
Peak AU 17	–0.15	–0.02	0.07	**0**.**38**	–0.03	**0**.**39**	0.13	0.13	0.13
Peak AU 23	–0.17	0.05	0.17	**0**.**37**	–0.23	0.28	–0.05	0.03	–0.12
Peak AU 24	–0.08	0.04	0.07	0.28	–0.23	0.27	–0.01	0.15	–0.08
Peak AU 25	**0**.**53**	**0**.**53**	–0.07	**–0**.**34**	0.18	**–0**.**38**	0.11	0.07	–0.04
Peak AU 43	0.27	–0.21	–0.04	–0.11	–0.21	0.11	0.19	–0.08	0.03

Correlations > 0.30 are in bold. PI, purchase intention, AU04 = brow lowerer, AU05 = upper, lid raiser, AU06 = cheek raiser, AU07 = lid tightener, AU12 = lip corner pull, AU14 = dimpler, AU15 = lip corner depressor, AU17 = chin raiser, AU23 = lip tightener, AU24 = lip pressor, AU25 = lips part, AU43 = eyes closed. Ad like, Advertisement likeability; Brand like, Brand likeability change; Purchase intention, brand purchase intention change.

### Semiparametric models of AU activity

We fitted separate semiparametric additive mixed models to account for non-linear relationships while controlling for gender and stimuli as fixed factors and the participants and stimulus group as random factors. In the first step, we calculated regression models exclusively based on self-report ratings to test for specific relationships proposed by the affect-transfer hypothesis (see Table 2). It is evident that advertisement likeability is strongly predicted by joy ratings, brand likeability change is only significantly predicted by advertisement likeability, and purchase intention change is strongly predicted by brand likeability. This pattern strongly supports a hierarchical influence of advertisement and brand effects as postulated by the affect-transfer hypothesis.

**TABLE 2:** Prediction of ad likeability, brand likeability change, and brand purchase intention change based on relevant self-report ratings.

	Ad like			Brand like			Purchase intention		
	**df**	***F***	***p***	**df**	***F***	***p***	**df**	***F***	***p***
Brand like							**3.39**	**124.62**	**<0.001**
Ad like				**1.58**	**72.64**	**<0.001**	1.58	2.23	0.072
Joy	**5.26**	**185.8**	**<0.001**	1.00	3.39	0.066	**2.80**	**2.38**	**0.045**
*R* ^2^_adj_	0.445			0.141			0.262		
*AIC*	3,969			4,891			4,646		
*BIC*	4,351			5,285			5,051		

Significant coefficients are in bold. The models are controlled for gender and stimuli as fixed factors and participants and stimulus groups as random factors. Ad like, Advertisement likeability; Brand like, Brand likeability change; Purchase intention, brand purchase intention change.

Next, we fitted separate models to predict joy ratings (Table 3), advertisement likeability (Table 4), brand likeability change (Table 5), and purchase intention change (Table 6) based on mean and peak AU activity. In addition, we estimated the combined effect of AU and rating scales to determine the relative predictiveness of AFC versus self-report for the models that contain advertisement and brand effect self-report ratings.

**TABLE 3:** Prediction of joy ratings based on mean and peak Action Unit (AU) activity.

	Joy					
	**AU mean**			**AU peak**		
	**df**	***F***	***p***	**df**	***F***	***p***
AU04	1.51	1.24	0.431	1.00	0.07	0.787
AU05	1.42	0.13	0.747	1.51	0.21	0.847
AU06	**1.00**	**9.39**	**0.002**	**1.00**	**6.70**	**0.010**
AU07	1.00	0.25	0.617	1.31	0.64	0.403
AU12	**4.27**	**15.09**	**<0.001**	**1.21**	**34.27**	**<0.001**
AU14	1.00	0.21	0.644	3.70	1.13	0.321
AU15	1.00	1.69	0.194	1.03	1.73	0.188
AU17	1.00	0.01	0.939	1.06	0.06	0.859
AU23	1.00	0.39	0.534	1.00	1.90	0.168
AU24	1.00	0.04	0.841	1.55	0.66	0.340
AU25	2.47	2.53	0.125	1.37	0.78	0.357
AU43	1.00	0.80	0.373	1.71	0.66	0.561
*R* ^2^ *_adj_*	0.373			0.373		
*AIC*	4,249			4,244		
*BIC*	4,752			4,748		

Significant coefficients are in bold. The models are controlled for gender and stimuli as fixed factors and participants and stimulus groups as random factors. AU04 = brow lowerer, AU05 = upper, lid raiser, AU06 = cheek raiser, AU07 = lid tightener, AU12 = lip corner pull, AU14 = dimpler, AU15 = lip corner depressor, AU17 = chin raiser, AU23 = lip tightener, AU24 = lip pressor, AU25 = lips part, AU43 = eyes closed.

**TABLE 4:** Prediction of advertisement likeability ratings based on mean and peak Action Unit (AU) activity with and without self-report ratings.

	Ad like ratings											
	**AU mean**			**AU mean + ratings**			**AU peak**			**AU peak + ratings**		
	**df**	***F***	***p***	**df**	***F***	***p***	**df**	***F***	***p***	**df**	***F***	***p***
Joy				**5.27**	**152.66**	**<0.001**				**5.69**	**135.39**	**<0.001**
AU04	1.00	3.53	0.060	1.00	1.99	0.159	**1.00**	**6.87**	**0.009**	**1.06**	**7.67**	**0.005**
AU05	1.00	0.01	0.932	1.00	0.08	0.778	1.29	0.06	0.818	2.53	1.94	0.187
AU06	**1.00**	**13.95**	**<0.001**	**1.00**	**4.46**	**0.035**	**1.00**	**10.33**	**0.001**	2.17	2.78	0.059
AU07	1.00	1.35	0.245	1.00	2.75	0.097	1.00	1.45	0.229	1.14	3.51	0.058
AU12	**4.89**	**17.96**	**<0.001**	**1.59**	**6.74**	**0.002**	**2.11**	**29.22**	**<0.001**	**1.00**	**9.31**	**0.002**
AU14	2.72	1.69	0.124	**2.70**	**3.00**	**0.033**	1.00	0.17	0.679	1.11	0.17	0.757
AU15	1.00	0.00	0.954	1.00	1.74	0.188	1.32	0.34	0.509	1.72	0.43	0.671
AU17	1.00	0.13	0.722	1.00	0.20	0.655	1.51	0.15	0.827	1.10	0.30	0.559
AU23	1.13	0.04	0.859	1.00	0.07	0.796	1.00	1.04	0.307	1.95	0.67	0.541
AU24	1.00	1.12	0.290	1.00	1.23	0.268	1.00	1.21	0.271	1.48	0.23	0.838
AU25	1.00	1.24	0.266	**1.00**	**4.98**	**0.026**	1.48	2.03	0.234	1.81	2.04	0.191
AU43	1.00	0.00	0.968	1.00	0.14	0.704	1.00	0.01	0.926	1.72	0.41	0.634
*R* ^2^ *_adj_*	0.250			0.455			0.247			0.457		
*AIC*	4,652			4,038			4,643			4,042		
*BIC*	5,155			4,552			5,147			4,556		

Significant coefficients are in bold. The models are controlled for gender and stimuli as fixed factors and participants and stimulus groups as random factors. AU04 = brow lowerer, AU05 = upper, lid raiser, AU06 = cheek raiser, AU07 = lid tightener, AU12 = lip corner pull, AU14 = dimpler, AU15 = lip corner depressor, AU17 = chin raiser, AU23 = lip tightener, AU24 = lip pressor, AU25 = lips part, AU43 = eyes closed. Ad like, Advertisement likeability.

**TABLE 5:** Prediction of brand likeability change ratings based on mean and peak Action Unit (AU) activity with and without self-report ratings.

	Brand like change ratings											
	**AU mean**			**AU mean + ratings**			**AU peak**			**AU peak + ratings**		
	**df**	***F***	***p***	**df**	***F***	***p***	**df**	***F***	***p***	**df**	***F***	***p***
Ad like				**1.59**	**67.95**	**<0.001**				**1.22**	**82.76**	**<0.001**
Joy				1.00	3.11	0.078				1.00	2.51	0.114
AU04	1.00	0.00	0.998	1.00	0.43	0.511	**1.00**	**10.78**	**0.001**	**1.00**	**7.43**	**0.006**
AU05	1.00	0.00	0.961	1.00	0.03	0.861	1.14	0.03	0.958	1.00	0.07	0.793
AU06	1.00	0.01	0.938	1.41	0.68	0.360	2.09	1.91	0.135	2.11	2.11	0.101
AU07	1.00	0.07	0.794	1.00	0.04	0.833	1.00	0.89	0.347	1.00	0.36	0.550
AU12	**1.90**	**4.57**	**0.007**	1.00	1.39	0.240	**2.31**	**4.06**	**0.012**	2.45	1.21	0.400
AU14	2.40	1.83	0.100	2.05	1.08	0.307	1.00	0.08	0.778	1.00	0.18	0.672
AU15	1.00	0.55	0.457	1.00	0.75	0.387	1.00	0.17	0.680	1.00	0.05	0.830
AU17	1.00	0.41	0.521	1.00	0.68	0.410	1.00	0.88	0.349	1.00	0.83	0.363
AU23	2.11	1.19	0.255	1.96	1.25	0.339	1.84	0.89	0.336	1.87	0.96	0.358
AU24	1.00	1.02	0.313	1.32	0.40	0.497	1.00	0.37	0.541	1.00	0.10	0.750
AU25	1.00	0.00	0.965	1.00	0.15	0.701	1.00	0.08	0.773	1.00	0.01	0.917
AU43	1.00	0.02	0.882	1.00	0.00	0.995	1.14	0.26	0.733	1.34	0.35	0.756
*R* ^2^ *_adj_*	0.057			0.142			0.064			0.146		
*AIC*	5,137			4,995			5,121			4,988		
*BIC*	5,640			5,520			5,625			5,513		

Significant coefficients are in bold. The models are controlled for gender and stimuli as fixed factors and participants and stimulus groups as random factors. AU04 = brow lowerer, AU05 = upper, lid raiser, AU06 = cheek raiser, AU07 = lid tightener, AU12 = lip corner pull, AU14 = dimpler, AU15 = lip corner depressor, AU17 = chin raiser, AU23 = lip tightener, AU24 = lip pressor, AU25 = lips part, AU43 = eyes closed. Ad like, Advertisement likeability; Brand like, Brand likeability change.

**TABLE 6:** Prediction of brand purchase intention change rating based on mean and peak Action Unit (AU) activity with and without self-report ratings.

	Purchase intention											
	**AU mean**			**AU mean + ratings**			**AU peak**			**AU peak + ratings**		
	**df**	***F***	***p***	**df**	***F***	***p***	**df**	***F***	***p***	**df**	***F***	***p***
Brand like				**4.18**	**100.38**	**<0.001**				**3.58**	**117.77**	**<0.001**
Ad like				1.75	2.23	0.070				**1.71**	**2.60**	**0.049**
Joy				**2.79**	**2.60**	**0.035**				**2.85**	**2.69**	**0.031**
AU04	2.36	2.27	0.205	2.04	2.22	0.128	1.39	1.09	0.228	1.34	0.15	0.855
AU05	1.00	0.78	0.376	1.03	1.30	0.260	1.00	0.25	0.621	1.00	0.26	0.613
AU06	1.00	0.28	0.597	1.00	0.92	0.337	1.00	0.15	0.701	1.00	0.00	0.998
AU07	1.00	0.97	0.325	1.00	0.81	0.368	1.39	0.49	0.689	1.40	0.21	0.816
AU12	1.43	0.56	0.375	1.00	0.55	0.459	1.00	0.71	0.399	1.59	1.48	0.206
AU14	**2.63**	**6.28**	**<0.001**	**1.73**	**8.49**	**0.003**	1.00	1.82	0.178	1.51	2.21	0.231
AU15	1.52	0.18	0.780	2.22	1.07	0.518	1.00	0.01	0.905	1.00	0.44	0.507
AU17	1.00	2.56	0.110	1.00	1.96	0.162	1.16	0.04	0.860	1.00	0.74	0.391
AU23	1.53	0.27	0.719	1.00	0.40	0.527	1.00	0.24	0.625	1.00	0.00	0.959
AU24	**1.00**	**4.39**	**0.036**	1.00	3.04	0.081	1.00	0.07	0.798	1.00	0.78	0.377
AU25	1.00	1.26	0.262	1.54	1.53	0.361	1.00	0.36	0.550	1.00	0.38	0.538
AU43	1.43	2.39	0.068	**1.00**	**4.88**	**0.027**	1.69	2.04	0.257	1.48	1.78	0.298
*R* ^2^ *_adj_*	0.055			0.275			0.034			0.263		
*AIC*	5,151			4,733			5,175			4,752		
*BIC*	5,654			5,269			5,678			5,288		

Significant coefficients are in bold. The models are controlled for gender and stimuli as fixed factors and participants and stimulus groups as random factors. AU04 = brow lowerer, AU05 = upper, lid raiser, AU06 = cheek raiser, AU07 = lid tightener, AU12 = lip corner pull, AU14 = dimpler, AU15 = lip corner depressor, AU17 = chin raiser, AU23 = lip tightener, AU24 = lip pressor, AU25 = lips part, AU43 = eyes closed. Ad like, Advertisement likeability; Brand like, Brand likeability change; Purchase intention, brand purchase intention change.

#### Prediction of joy ratings

**FIGURE 2:**
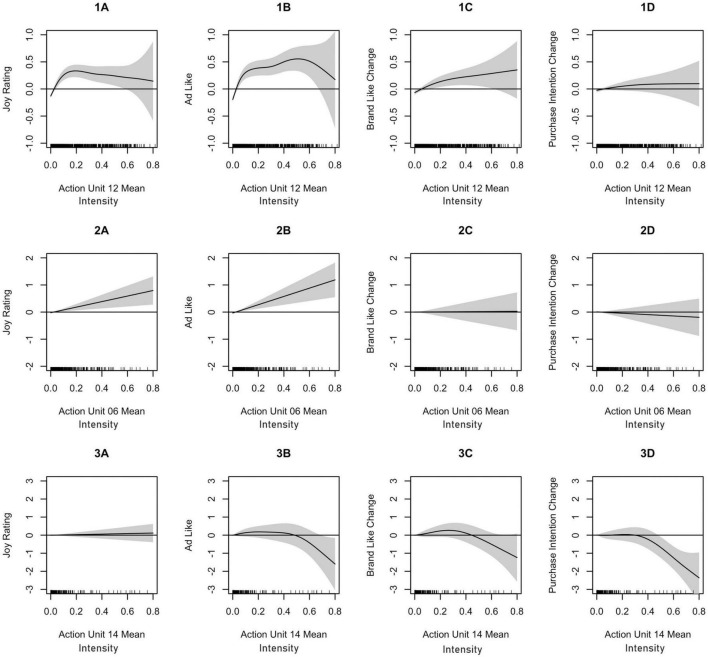
Fitted smooth main effects model for Action Unit means of AU12 [lip corner pull, **(1A–1D)**], AU06 [cheek raiser, **(2A–2D)**], and AU14 [dimpler, **(3A–3D)**]. The graphs show the estimated marginal effects on joy ratings **(1A–3A)**, advertisement likeability **(1B–3B)**, brand likeability change **(1C–3C)**, and purchase intention change **(1D–3D)**. The effects are centered around zero. The shaded areas show 95% pointwise confidence intervals.

**FIGURE 3:**
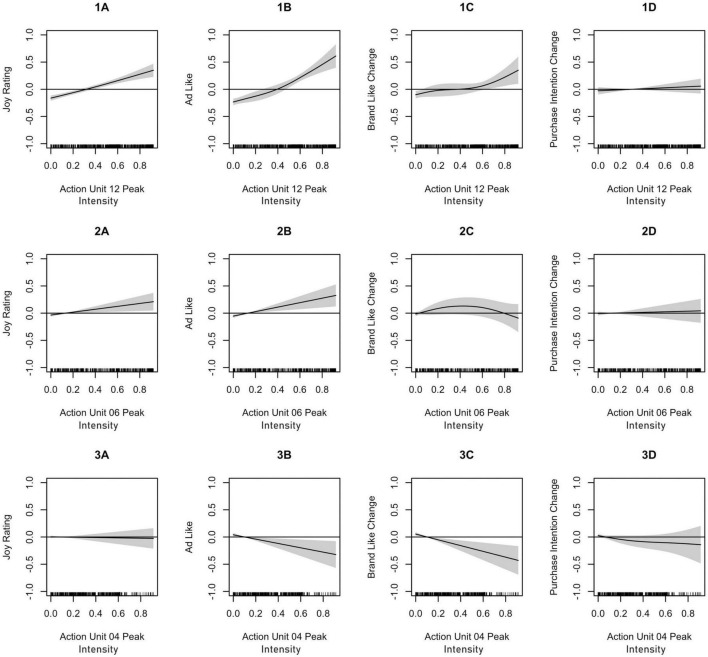
Fitted smooth main effects model for Action Unit peaks of AU12 [lip corner pull, **(1A–1D)**], AU06 [cheek raiser, **(2A–2D)**], and AU04 [brow raiser, **(3A–3D)**]. The graphs show the estimated marginal effects on joy ratings **(A)**, advertisement likeability **(B)**, brand likeability change **(C)**, and purchase intention change **(D)**. The effects are centered around zero. The shaded areas show 95% pointwise confidence intervals.

Joy ratings were significantly predicted by mean and peak activities of AU6 and AU12 (see Table 3). Mean AU12 (Figure 2, Panel 1A) showed a non-linear association with joy ratings, with the highest values for moderate AU intensities. In contrast, AU12 peak (Figure 3, Panel 1A), AU6 mean (Figure 2, Panel 2A), and AU6 peak (Figure 3, Panel 2A) activity showed a linear and strictly monotonically increasing function with regard to joy ratings.

#### Prediction of advertisement likeability

Advertisement likeability ratings were significantly predicted by mean activity of AU6 and AU12 as well as peak activity of AU4, AU6, and AU12 (see Table 4). Mean AU12 (Figure 2, Panel 1B) showed a non-linear association with advertisement likeability ratings, with the highest values for moderate AU intensities. In contrast, AU12 peak (Figure 3, Panel 1B), AU6 mean (Figure 2, Panel 2B), AU6 peak (Figure 3, Panel 2B) showed a linear and strictly monotonically increasing function, whereas AU4 peak activity (Figure 3, Panel 3B) showed a linear and strictly monotonically decreasing function with regard to advertisement ratings. These effects remained still significant for most AUs if joy ratings were included in the models.

#### Prediction of brand likeability change

Brand likeability change ratings were significantly predicted by mean and peak activity of AU12 as well as peak activity of AU4 (see Table 5). AU12 mean (Figure 2, Panel 1C) and AU12 peak (Figure 3, Panel 1C) showed strictly monotonically increasing functions, whereas AU4 peak activity (Figure 3, Panel 3C) showed a strictly monotonically decreasing function with regard to brand likeability change ratings. These effects for brand likeability change ratings remained only significant for peak AU4 activity if joy and advertisement likeability ratings are included in the models.

#### Prediction of purchase intention change

Purchase intention change ratings were significantly predicted by mean activity of AU14 and AU 24 (see Table 6). AU14 mean (Figure 2, Panel 3D) and AU24 mean activity showed strictly monotonically decreasing functions with regard to purchase intention change ratings. These effects for purchase intention change ratings remained only significant for mean AU14 activity if joy, advertisement likeability, and brand likeability change ratings are included in the model.

#### Variation in model fit

Notably, there was a large variation in model fit (i.e., adjusted explained variance), which depends on the specific criterium and whether self-report rating scales are included in the model in addition to the facial expression parameters. For models including only AU parameters, we observed that the explanation of variance was strong for emotion ratings of joy, moderate to strong for advertisement likeability, and small for brand effects such as likeability and purchase intention change. If AU parameters and relevant rating scales are jointly used, we were able to improve model fit significantly and found a strong variance explanation for advertisement likeability mainly driven by joy ratings, a moderate variance explanation for brand likeability change mainly driven by advertisement likeability ratings, and a strong variance explanation for purchase intention change mainly driven by brand likeability change. Taken together, we demonstrated that AU parameters predicted relevant advertisement and brand criteria beyond self-report (see also Table 7).

**TABLE 7:** Predictions of emotional processes according to a universalist (Cordaro et al., 2018), an appraisal-driven approach (Scherer et al., 2018), and a summary of observations in the present study for the relevant subset of Action Units (AU).

AU	AU description	Cordaro et al., 2018	Scherer et al., 2018	Present findings
AU4	Brow lowerer	Anger, confusion, disgust, pain, shame, sadness, and contempt	Novelty, unpleasant, goal obstructive, and high coping potential	Lower ad like (peak), lower brand like (peak)
AU6	Cheek raiser	Amusement, triumph, joy, desire, coyness, embarassment, disgust, and pain		Higher joy (mean + peak), higher ad like (mean + peak)
AU12	Lip corner pull	Amusement, triumph, joy, desire, coyness, embarassment, pride, content, relief, and awe	Pleasant, goal conductive	Higher joy (mean + peak), higher ad like (mean + peak), higher brand like (mean + peak)
AU14	Dimpler	Contempt		Lower ad like (mean) lower purchase intention (mean)
AU24	Lip pressor		Unpleasant, high coping potential	Lower purchase intention (mean)
AU25	Lips part	Amusement, triumph, joy, coyness, relief, embarassment; disgust, pain, sympathy, contempt, fear, awe, surprise	Pleasant, unpleasant, goal conductive, high coping potential, low coping potential	Higher ad like (mean)
AU43	Eyes closed	Content, relief; pain, sadness, contempt, and boredom	Unpleasant, high coping potential	Lower purchase intention (mean)

## Discussion

Commercials are thought to elicit emotions, but it is difficult to quantify viewers’ emotional responses objectively and whether this has the intended impact on consumers. With novel technology, we automatically analyzed facial expressions in response to a broad array of video commercials and predicted self-reports of advertisement and brand effects. Taken together, parameters extracted by an automated facial coding technology significantly predicted all dimensions of self-report measures. Hence, automatic facial coding can contribute to a better understanding of advertisement effects in addition to self-report.

However, there was also a tremendous difference in model fit and, particularly, the strength of effects: facial expressions predicted emotion ratings with strong effects, advertisement likeability with moderate effects, changes in brand likeability, and purchase intention only with small effects. Furthermore, relations between self-report ratings strongly support a hierarchical influence of advertisement and brand effects as postulated by the affect-transfer-hypothesis: We found strong associations between joy and advertisement likeability, moderate effects between advertisement likeability and changes in brand likeability and again strong relations between brand likeability and change in purchase intention elicited by video commercials intention. Accordingly, AFC might be a valid indicator for measuring joy experience, advertisement, and brand effects, but the relevant self-report dimension still predicted the investigated criteria with more substantial effects.

Table 7 summarizes the specific effects of mean and peak AU activity on the investigated criteria. AU12 (lip corner pull) compared to other AU was a significant predictor of joy experience, ad likeability, and brand likeability change, which is in line with previous research (Lewinski et al., 2014b; McDuff et al., 2014, 2015). In contrast to the previous report (Teixeira et al., 2014; McDuff et al., 2015), we observed no significant relationship between AU12 and purchase intention. However, we found facial activity related to unpleasant emotional states to predict purchase intention, such as AU14 (Dimpler), AU24 (Lip Pressor), and AU43 (Eyes Closed). Such contradicting results may be explained by different operationalization of the purchase intention across studies: While previous literature measured purchase intention only per post-advertisement measures (Lewinski et al., 2014b; McDuff et al., 2014, 2015; Teixeira et al., 2014), in the present study this construct was measured by pre to post changes. This is preferable because post-advertisement brand ratings are highly confounded with the selected brand stimuli and cannot validly measure the effects of commercials. Hence, we found several Action Unit patterns that were not investigated before, expanding our knowledge of the relation between facial expressions and advertisement and brand effects.

The presented findings also contribute to a better understanding of the relationships of different statistical aggregation strategies of AFC parameters. In particular, the experience and memorization of emotional events is influenced or even biased by different aggregates of such a dynamic time-series (Fredrickson, 2000). Specifically, we analyzed mean and peak AU activities which are both widely used aggregates in emotion research. Importantly, we found coherence and exclusive contributions of peak and mean statistics. Mean and peak statistics of AU06 (cheek raiser) and AU12 (lip corner pull) show no meaningful differences in the prediction of joy ratings. However, predictions of advertisement likeability and brand likeability change also demonstrate a differential impact of specific AU patterns. For example, peak activity of AU04 (Brow Lowerer) had a significant effect on these criteria, which was not the case for mean values of AU04. Hence, our findings contribute to a better understanding of the differential impact of facial expression aggregates in advertisement and brand research. Future studies should investigate the role of other associated phenomena, such as the peak-end-bias (e.g., Do et al., 2008) and the stability of effects over time in advertisement research.

### Limitations and future directions

One aspect of the present study is the exclusive use of self-report ratings as the criteria in a cross-sectional design. The usage of *ad hoc* self-report scales has two significant limitations: First, it is unclear how stable reported advertisement effects on relevant brand dimensions are over time. Long-term effects of advertisement might be explored through longitudinal study design in future research, for example, by inviting participants again weeks or months after the main experiment to probe the stability of brand likeability and purchase intention changes. Second, it is unclear whether psychologically assessed intentions to purchase products or services of a particular brand elicit an actual purchase behavior. Future research should focus on predictions of actual behavior or even population-wide effects like it is approached with other methods in the consumer neuroscience literature (Berkman and Falk, 2013). Hence, out-of-sample criteria in the consumer research area that facial expression parameters might predict advertisement effects on a market-level response, such as monetary advertising elasticity estimates (Venkatraman et al., 2015) or video view frequencies on media platforms (Tong et al., 2020). Furthermore, facial responses toward music and movie trailers could predict actual sales figures in the music and movie industry, as already demonstrated with measures of neural response (Berns and Moore, 2012; Boksem and Smidts, 2015). Hence, future research needs to explore the predictive capability of facial expression recognition technology beyond within-subject measured self-report.

Automatic facial coding has also some advantages in comparison to emotional self-report because it enables a passive and non-contact assessment of emotional responses on a continuous basis. In contrast, emotional self-report is typically rated after stimulus presentation, and hence, reflect a more global and possibly biased evaluation of the recipients (e.g., Müller et al., 2019). Furthermore, AFC provides a rich data stream of emotion-relevant facial movements, whereas self-report is typically assessed on a limited number of emotion scales. AFC technology enables a moment-to-moment analysis of elicited emotional responses, which allows for the assessment of emotional responses toward dynamic emotional content as in video commercials. For example, stories can have very different emotional dynamics such as an unpleasant beginning and a pleasant end and vice versa (Reagan et al., 2016). Hence, future research should investigate the differential impact of emotional dynamics of advertisement commercials and whether differences in the emotional dynamics affect relevant advertisement and brand effects.

## Conclusion

The present study identified facial expressions that were validated by self-reported emotional experience and predicted changes in brand likeability and purchase intention. Hence, this novel technology may be an excellent tool for tracking advertisement effects in real-time. Automatic facial coding enables a moment-to-moment analysis of emotional responses, non-invasive and non-contact. Accordingly, automatic emotional facial expression recognition is suitable for advertisement optimization based on emotional responses and for online research. Future research needs to evaluate the capability of such technology to predict actual consumer behavior beyond self-report and with out-of-sample criteria. Facial expressions can reveal very private emotional states and there will probably be a remarkable increase in the use of face recognition technology and its integration in everyday situations. Consequentially, many ethical issues will arise, specifically if applied in commercial and political contexts, and in particular if facial information is collected or analyzed without consent.

## Data availability statement

The datasets presented in this study can be found in online repositories. The names of the repository/repositories and accession number(s) can be found below: https://madata.bib.uni-mannheim.de/id/eprint/410.

## Ethics statement

The studies involving human participants were reviewed and approved by the Research Ethics Committee of the University of Mannheim. The patients/participants provided their written informed consent to participate in this study.

## Author contributions

TH contributed to the conception and design of the study, collected the data, performed the statistical analysis, and wrote the first draft of the manuscript. TH and GA reviewed and approved the final manuscript.
