# Supplementary material for: Automatic facial coding predicts self-report of emotion, advertisement and brand effects elicited by video commercials
Source: Front Neurosci. 2023 May 2;17:1125983. doi: 10.3389/fnins.2023.1125983 (PMC10185761; doi:10.3389/fnins.2023.1125983)
Supplement: Supplementary file 1 [file Data_Sheet_1.pdf]

Appendix 1: Advertisement stimuli sorted by stimulus groups and corresponding mean emotion and familiarity ratings

Group	Title	Brand	Dur-ation	Industry	Familiari-ty Brand	Familiar-ity Video	Joy Rating	Sadness Rating	Anger Rating	Fear Rating	Disgust Rating	Surprise Rating
A1	Daughter	Audi	01:00	automotive	8.96	1.38	5.77	1.65	1.73	1.38	1.04	4.19
A2	Year in search 2016	Google	02:00	electronic	9.00	1.31	6.19	4.85	3.69	2.73	1.92	4.27
A3	Unlimited Future	Nike	01:19	fashion	8.96	1.31	6.42	1.73	1.62	1.31	1.08	5.19
A4	Mechanic	Cilit Bang	01:37	fmcg	8.73	2.73	7.42	1.08	1.31	1.00	1.31	4.96
A5	Be the fastest	Virgin Media	01:40	media	3.35	1.31	6.04	1.12	1.69	1.23	1.35	3.38
A6	Forest	Geiko	01:26	ensurance	2.12	1.31	7.42	1.58	1.58	1.38	1.12	5.46
A7	Snaples commercial	Snaples	00:30	fmcg	2.81	1.27	5.62	1.19	1.46	1.27	1.69	5.12
A8	Man On The Moon	John Lewis	02:06	retail	3.54	1.31	6.15	5.31	1.31	1.73	1.12	4.88
B1	Titans	Nissan	01:30	automotive	8.90	1.28	4.14	2.52	1.79	1.62	1.28	4.10
B2	Odds	Adidas	01:39	fashion	9.00	1.07	4.07	3.83	1.97	2.00	1.83	4.14
B3	Wiener stempede	Heinz	01:00	fmcg	7.38	1.72	6.69	1.28	2.00	1.17	1.97	4.69
B4	Bulbs	Apple	01:37	electronic	8.97	1.48	4.97	1.14	2.69	1.45	1.21	5.62
B5	Lily and the snowman	Cineplex	02:03	media	7.28	1.31	7.03	4.38	1.55	1.66	1.17	3.97
B6	Tiny Dancer	John Lewis	01:30	ensurance	3.10	1.55	7.07	1.52	1.52	1.69	1.10	4.79
B7	Tide's Super Bowl Stain	P&G Tide	01:15	fmcg	7.62	1.31	5.31	1.24	1.52	1.21	1.38	4.52
B8	Blood	Libresse	01:00	fmcg	1.90	1.14	2.66	1.93	2.97	2.34	5.28	4.72
C1	david beckham essentials	H&M	01:22	fashion	8.97	1.45	5.84	1.26	1.55	1.23	1.13	4.26
C2	Alien	VW	01:02	automotive	8.97	1.06	4.97	1.68	2.00	3.35	4.52	5.32
C3	Liam	Apple	00:59	electronic	9.00	1.65	4.00	1.06	1.29	1.06	1.03	3.26
C4	The Letter	Otto	01:22	retail	8.55	2.03	5.94	5.32	1.35	1.32	1.03	4.03
C5	Protect Like a Mother	Lysol	01:00	fmcg	1.42	1.26	6.32	1.65	1.55	1.35	1.13	5.23
C6	Buster the boxer	John Lewis	02:00	retail	3.13	2.23	7.00	1.97	1.19	1.16	1.10	4.84
C7	Rocket car	Old Spice	01:00	fmcg	5.00	1.39	4.06	1.61	1.58	1.55	1.26	4.81
C8	Jeff and his forest friends	Johnsonville	01:00	fmcg	1.45	1.23	6.03	1.35	1.55	1.19	1.52	4.16
D1	You're alive	Hornbach	01:00	retail	8.59	4.37	6.59	1.22	1.11	1.19	2.89	4.30
D2	Your future is not mine	Adidas	01:15	fashion	8.63	1.59	3.74	2.78	1.89	2.30	1.89	2.93
D3	Marylin	Snickers	00:30	fmcg	8.96	2.41	6.30	1.07	1.30	1.04	1.81	4.63
D4	The Billion Color Film	Samsung	01:45	electronic	9.00	1.67	3.89	2.67	1.78	1.56	1.19	3.37
D5	The corner	Powerade	02:00	fmcg	6.04	1.37	2.52	3.22	3.26	1.93	2.19	2.63
D6	Rule yourself	Under Armor	01:23	fashion	5.15	1.96	3.48	2.37	1.41	1.93	1.52	2.44
D7	Conference	Loctite	00:20	fmcg	1.52	1.63	5.04	1.00	1.07	1.00	1.00	4.63
D8	Strong	P&G	02:00	fmcg	7.52	2.59	6.22	3.85	1.41	1.63	1.07	3.85

Appendix 1 (continued)

Group	Title	Brand	Dur-ation	Industry	Familiari-ty Brand	Familiar-ity Video	Joy Rating	Sadness Rating	Anger Rating	Fear Rating	Disgust Rating	Surprise Rating
E1	Stroll	Apple	01:00	electronic	8.47	2.67	7.13	1.27	1.30	1.17	1.03	3.23
E2	Benz Kids	Daimler	00:45	automotive	8.87	2.80	7.63	1.07	1.40	1.00	1.00	5.33
E3	The Conductor	Nike	01:30	fashion	8.90	1.00	5.60	1.97	2.00	1.37	1.50	4.33
E4	We are awake	Mc Donalds	01:00	fmcg	8.50	1.80	4.73	2.13	1.67	1.07	1.57	2.63
E5	Christmas with love from Mrs. Claus	Marks and Spencer	01:58	retail	3.77	1.27	6.97	3.33	1.63	1.37	1.33	4.63
E6	Making Water Active	G Active	01:00	fmcg	2.70	1.27	4.47	1.17	1.47	1.17	1.23	3.80
E7	Mailshrimp	Mailchimp	01:00	electronic	1.20	1.00	5.47	1.37	1.70	1.03	2.77	4.03
E8	Sarah & Juan	Weekly Extra	01:58	fmcg	8.67	2.23	7.30	4.10	1.17	1.40	1.13	4.40
F1	Laughing Horses	VW	00:47	automotive	8.93	3.15	7.15	1.15	1.52	1.15	1.37	4.26
F2	Ostrich	Samsung	01:49	electronic	8.96	3.22	7.67	1.41	1.19	1.19	1.19	4.22
F3	Pool Boy	Coca-Cola	01:30	fmcg	8.70	3.30	6.04	1.19	1.52	1.15	1.59	3.93
F4	Heimkommen	Edeka	01:46	retail	8.85	7.52	4.89	7.56	2.96	2.67	1.70	5.04
F5	Bullish Boss	Clear	00:30	fmcg	2.00	1.04	2.56	1.59	2.96	3.56	4.70	5.93
F6	Question Madness	North Face	01:45	fashion	7.67	1.19	5.07	2.04	2.07	3.48	2.30	2.74
F7	Canal Kitchen	Canal+	01:00	electronic	2.85	1.04	5.00	1.41	1.78	1.30	1.78	5.07
F8	The ring	Pot Noodle	01:00	fmcg	2.19	1.26	5.89	1.48	1.89	1.22	2.89	6.81
G1	Go time	Apple	01:00	electronic	8.96	2.21	4.29	1.17	1.58	1.29	1.17	1.75
G2	Forever chuck	Converse	02:00	fashion	8.96	1.00	3.42	1.33	2.21	1.75	1.42	3.46
G3	Original is never finished	Adidas	01:30	fashion	8.92	1.33	3.29	2.50	2.04	2.33	2.00	4.46
G4	Hero‘s journey	Kia Niro	01:01	automotive	8.63	1.33	6.79	1.33	1.88	1.42	1.21	4.79
G5	Anthony Joshua	Lucozade	01:30	fmcg	1.00	1.00	4.00	2.29	1.96	1.46	1.00	3.96
G6	The Swim	Klarna	00:30	electronic	4.42	1.25	5.58	1.33	1.54	1.33	1.38	4.92
G7	Food for men	Stryhns	00:30	fmcg	1.08	2.13	6.96	1.50	1.92	1.04	1.29	4.92
G8	Coming home	Waitrose	01:30	retail	1.04	1.00	6.79	3.67	1.50	2.71	1.00	4.42
H1	Equality	Nike	01:30	fashion	8.84	1.32	4.96	3.40	2.44	1.60	1.04	2.32
H2	Opening doors	VW	00:46	auto	8.68	1.12	5.28	1.64	2.00	2.56	2.52	6.60
H3	Free up space	Google	01:00	electronic	9.00	2.44	6.92	1.12	1.64	1.00	1.04	3.36
H4	Commander	Audi	01:30	automotive	8.88	4.56	6.32	3.28	1.40	1.28	1.04	3.76
H5	High school girl	Shiseido	02:27	fmcg	4.76	1.60	4.20	1.56	1.80	1.32	1.72	6.20
H6	Gender Violence	Tecate	01:17	fmcg	1.04	1.08	2.20	4.12	4.16	2.12	2.60	5.24
H7	Five Year Plan	Old Spice	00:47	fmcg	5.00	1.16	3.88	1.20	1.52	1.52	3.08	4.96
H8	Rimowa Electronic Tag	Rimowa	01:36	electronic	3.60	1.12	3.64	1.32	1.24	1.28	1.08	2.88

Appendix 2: Sample description (mean with standard deviation in brackets) and differences between groups

	Group A (n=26)	Group B (n=28)	Group C (n=31)	Group D (n=28)	Group E (n=30)	Group F (n=27)	Group G (n=24)	Group H (n=25)	Overall (n=219)	*p-value*
% females	50%	50%	52%	50%	53%	44%	67%	56%	53%	.887
STAI State	35.56 (6.21)	35.82 (6.71)	36.55 (5.61)	35.64 (4.94)	35.50 (6.06)	38.74 (5.80)	37.58 (8.05)	35.24 (6.27)	36.32 (6.22)	.404
STAI Trait	38.16 (6.55)	40.57 (7.44)	38.42 (8.70)	41.82 (9.88)	40.10 (8.23)	42.78 (10.51)	42.58 (11.75)	39.08 (10.99)	40.41 (9.35)	.425
SIAS	39.44 (9.39)	17.93 (10.59)	19.48 (11.39)	22.89 (10.60)	18.93 (8.76)	20.93 (15.58)	20.42 (11.64)	21.88 (11.14)	20.20 (11.20)	.776
BIS	15.44 (2.06)	15.75 (2.59)	15.84 (1.59)	14.71 (2.09)	15.17 (2.13)	15.33 (2.34)	14.63 (2.10)	15.32 (2.66)	15.29 (2.21)	.388
BAS	21.44 (4.11)	23.14 (4.13)	24.42 (4.47)	22.82 (5.14)	22.40 (4.43)	22.48 (6.59)	23.00 (4.93)	24.12 (4.79)	23.00 (4.88)	.399
PANAS PA	21.96 (3.70)	21.04 (3.28)	20.03 (4.21)	20.61 (3.22)	22.07 (4.78)	21.41 (3.30)	21.00 (4.54)	21.20 (4.10)	21.15 (3.93)	.554
PANAS NA	22.92 (3.48)	21.46 (3.46)	19.74 (4.37)	21.79 (3.40)	22.37 (5.03)	21.33 (3.50)	21.33 (5.10)	20.52 (3.49)	21.41 (4.09)	.111
BEQ	24.52 (3.43)	22.48 (4.10)	23.04 (4.18)	23.94 (3.96)	24.43 (3.57)	23.27 (3.57)	25.06 (3.59)	24.41 (4.01)	23.85 (3.85)	.194
SDS	35.52 6.24)	36.57 (6.52)	37.52 (6.92)	36.96 (5.48)	37.97 (5.40)	38.70 (6.32)	41.00 (9.00)	35.20 (8.96)	37.42 (6.99)	.089
